# Decomposition of spontaneous fluctuations in tumour oxygenation using BOLD MRI and independent component analysis

**DOI:** 10.1038/bjc.2015.270

**Published:** 2015-10-20

**Authors:** Miguel R Gonçalves, S Peter Johnson, Rajiv Ramasawmy, R Barbara Pedley, Mark F Lythgoe, Simon Walker-Samuel

**Affiliations:** 1 Centre for Advanced Biomedical Imaging, Division of Medicine, University College London, London WC1E 6DD, UK; 2 Cancer Institute, University College London, London WC1E 6DD, UK

**Keywords:** independent component analysis, pulse oximetry, systemic, tumour-specific, resting state

## Abstract

**Background:**

Solid tumours can undergo cycles of hypoxia, followed by reoxygenation, which can have significant implications for the success of anticancer therapies. A need therefore exists to develop methods to aid its detection and to further characterise its biological basis. We present here a novel method for decomposing systemic and tumour-specific contributions to fluctuations in tumour deoxyhaemoglobin concentration, based on magnetic resonance imaging measurements.

**Methods:**

Fluctuations in deoxyhaemoglobin concentration in two tumour xenograft models of colorectal carcinoma were decomposed into distinct contributions using independent component analysis. These components were then correlated with systemic pulse oximetry measurements to assess the influence of systemic variations in blood oxygenation in tumours, compared with those that arise within the tumour itself (tumour-specific). Immunohistochemical staining was used to assess the physiological basis of each source of fluctuation.

**Results:**

Systemic fluctuations in blood oxygenation were found to contribute to cycling hypoxia in tumours, but tumour-specific fluctuations were also evident. Moreover, the size of the tumours was found to influence the degree of systemic, but not tumour-specific, oscillations. The degree of vessel maturation was related to the amplitude of tumour-specific, but not systemic, oscillations.

**Conclusions:**

Our results provide further insights into the complexity of spontaneous fluctuations in tumour oxygenation and its relationship with tumour pathophysiology. These observations could be used to develop improved drug delivery strategies.

The structure of tumour vasculature is typically disorganised, with blood vessels that are tortuous, dilated, elongated and leaky, with saccular and dead-end formations (Carmeliet and Jain, 2000; Heldin *et al*, 2004). This vascular architecture, combined with long intervascular distances relative to the oxygen diffusion distance (Thomlinson and Gray, 1955), intravascular gradients of oxygen tension (Dewhirst *et al*, 1999) and increased blood viscosity (Kavanagh *et al*, 1993), contributes to the presence of regions of persistent low oxygen tension, or chronic hypoxia (Dewhirst, 1998).

Tumours can also exhibit regions with temporally variable oxygenation. This can be caused by phenomena such as arteriolar vasoconstrictive motion (vasomotion; Dewhirst *et al*, 1996), rapid vascular remodelling (Patan *et al*, 1996) and raised interstitial fluid pressure (Mollica *et al*, 2003), each of which can contribute to the observed spontaneous fluctuations in tumour blood flow and red blood cell flow (Kimura *et al*, 1996; Mollica *et al*, 2003). These are thought to be the cause of cycles of low oxygen tension with subsequent reoxygenation, a phenomenon known as cycling (or fluctuating) hypoxia (Chaplin *et al*, 1987; Kimura *et al*, 1996; Matsumoto *et al*, 2010).

Cycling levels of oxygenation have several significant implications for therapy. For example, they are a major cause of resistance to radiation therapy, owing to decreased number of reactive oxygen species (Martinive *et al*, 2006b) and reduced oxygen-induced stabilisation effect of DNA damage (Harrison and Blackwell, 2004). It also impacts negatively on the delivery of systematically administered chemotherapeutic agents, due to impairment of regional blood flow (which has further implications as the activity of some chemotherapeutic drugs is altered at low oxygen concentration; Durand, 2001; Chou *et al*, 2012). In addition, there is evidence that oxygen fluctuations can cause an increase in metastatic potential (Cairns *et al*, 2001). A deeper understanding of the tumour microenvironment and the influences behind these transient changes in oxygenation is therefore warranted.

Gradient-echo magnetic resonance imaging (GRE-MRI) sequences can be used to estimate blood oxygen variations non-invasively by mapping the blood oxygen level-dependent (BOLD) effect (Baudelet and Gallez, 2005). BOLD imaging uses the endogenous magnetic properties of deoxyhaemoglobin as a source of image contrast (Ogawa *et al*, 1990). Deoxyhaemoglobin is paramagnetic, and so an increase in deoxyhaemoglobin concentration can be detected in the decay of the GRE-MRI signal. This is quantified via the transverse relaxation rate (*R*_2_*=1/T_2_*). Consequently, blood oxygen fluctuations that are characteristic of cycling hypoxia can be estimated with measurements of temporal changes in *R*_2_* (Prasad *et al*, 1996). Gradient-echo imaging has been used in a number of studies to assess spontaneous cycling hypoxia events, which have aimed to identify the frequencies of such oscillations (Baudelet *et al*, 2004, 2006).

These fluctuations are complex and heterogeneously distributed throughout each tumour, and techniques to further evaluate and characterise them would allow the fluctuations to be better understood. To this end, we have adopted a technique that is now widely used in BOLD studies of the brain, named independent component analysis (ICA), which allows a signal to be decomposed into its constituent components. In the brain, ICA has been used to identify spatially coherent fluctuations in blood oxygenation that are associated with regions that are functionally connected (van de Ven *et al*, 2004; Beckmann *et al*, 2005; Kundu *et al*, 2013). By analogy, we propose that ICA could be used to identify regional connectivity within tumours, albeit reflecting different biological mechanisms to those in the brain, alongside the ability to study such signals in isolation, provide information on the sources of such fluctuations and investigate their relationship with variations in the tumour microenvironment. In this study, we provide an initial evaluation of the BOLD–ICA technique in two mouse xenograft models of colorectal carcinoma. We then use it in combination with pulse oximetry to identify contributions to tumour BOLD signal variations caused by changes in systemic blood oxygenation, thereby enabling fluctuations caused specifically by tumour pathophysiology to be isolated.

## Materials and Methods

### Animal models

**Figure 1:**
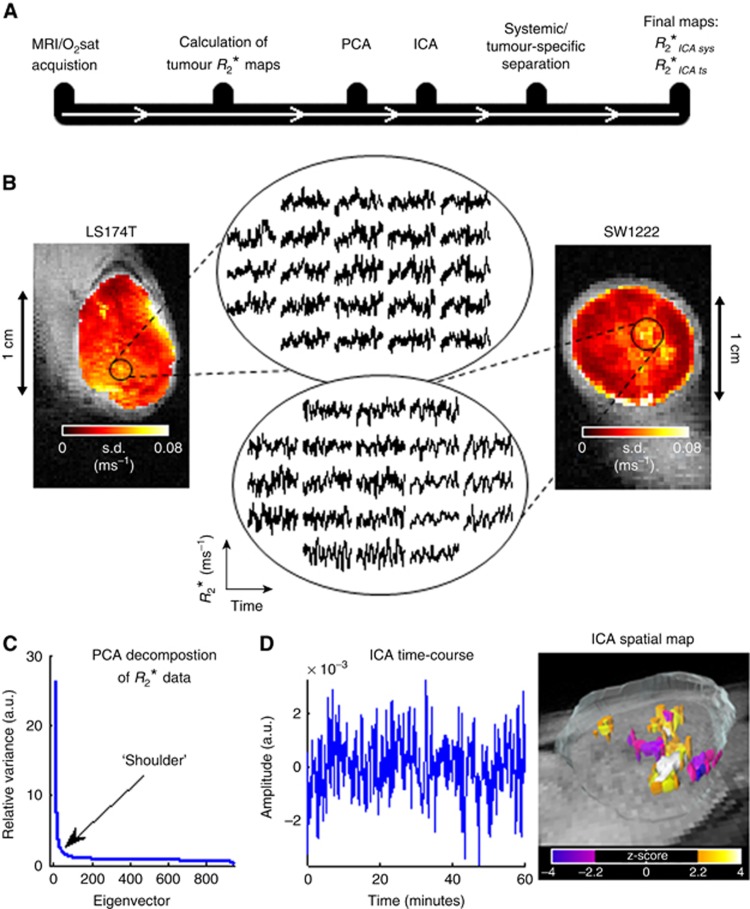
**Data acquisition and post-processing.** (**A**) Timeline of data acquisition and post-processing methods. (**B**) Voxel-wise standard deviation (s.d.) maps of the *R*_2_* fluctuations in example, representative, LS174T and SW1222 tumours, before any processing. s.d. maps are shown overlaid on T_2_-weighted images. High amplitude fluctuations, which are shown in the inset graphs, have larger s.d. values. (**C**) PCA decomposition curve showing the relative variance associated with each eigenvector. Eigenvectors to the left of the ‘shoulder' of the curve have high variance. (**D**) Time-course and 3D spatial map of one independent component in an example SW1222 tumour.

All experiments were performed in accordance with the local ethical review panel, the UK Home Office Animals Scientific Procedures Act 1986, and United Kingdom Co-ordinating Committee on Cancer Research guidelines (Workman *et al*, 2010). To propagate tumours, 5 × 10^6^ human colorectal adenocarcinoma cells (LS174T or SW1222) were injected subcutaneously into the flank of immunocompromised CD1 female nude mice (8 weeks of age, 20–25 g). These two cell lines develop into xenografts with markedly different phenotypes: SW1222 tumours are well-differentiated with clearly defined glandular structures and an extensive and well-perfused vascular network, whereas LS174T tumours show moderate-to-poor differentiation and are comparatively less-well vascularised and perfused, with larger hypoxic and necrotic regions (Pedley *et al*, 2002; El-Emir *et al*, 2005; Folarin *et al*, 2010). Mice were scanned at 2 weeks following inoculation, and tumour volumes ranged from 135 to 670 mm^3^. A summary of the data acquisition and post-processing is shown in Figure 1A.

### *In vivo* MRI

Mice bearing LS174T (*n*=5) or SW1222 (*n*=5) xenografts were anaesthetised using isoflurane in air and secured in a cradle using dental paste to minimise motion (Landuyt *et al*, 2002; Burrell *et al*, 2011). Warm air and water were used to maintain normothermia. Core temperature and respiratory rate were monitored using a rectal thermometer and a pressure pad, respectively (SA Instruments, Stony Brook, NY, USA). All MRI data were acquired using a horizontal bore 9.4 T scanner (Agilent Technologies, Santa Clara, CA, USA). Fast spin-echo (FSE) images were initially acquired for anatomical reference, from which we measured the tumour volume. We then acquired dynamic gradient-echo multi slice (GEMS) images, in order to map the temporal variation of the tumour relaxation rate, *R*_2_*. Imaging parameters for the FSE acquisition were: in-plane field of view 35 × 35 mm^2^, matrix size 128 × 128, slice thickness 1 mm, 20 slices. For the GEMS sequence we used: in-plane field of view 20 × 20 mm^2^, matrix size 64 × 64, slice thickness 1.5 mm, 5 slices, 3.8 s per image, total acquisition time for one animal was 60 min.

### *In vivo* pulse oximetry

During the acquisition of GEMS data, pulse oximetry recordings were obtained with a sensor clamped to the thigh of the animal (MouseOx, Starr Life Sciences, Oakmont, PA, USA), which allowed the direct measurement of systemic arterial oxyhaemoglobin saturation (O_2_sat).

### Model for MRI data decomposition

Tumour *R*_2_* time-courses (see Figure 1B, inset graphs) were first processed using principal component analysis (PCA), a computational technique that allows the extraction of the highest variance components in the data, and which is usually applied to highly dimensional data sets. In PCA, the variance in the data is associated to eigenvectors in a decreasing rank-order fashion, so that the first eigenvector has the largest variance, the second eigenvector has the second largest variance and so on (Shlens, 2014). This ordered process results in the last eigenvectors having very little variance, being thus ascribed to background noise (Shlens, 2014). Dimensionality reduction of the *R*_2_* data was then performed. This was achieved by selecting all the eigenvectors up to the ‘shoulder' of the PCA decomposition curve (see Figure 1C), as well as the eigenvectors after that point which were associated with a time-course exhibiting a dominant frequency peak. On average, 89 out of 944 eigenvectors per data set were chosen (*n*=10). This pre-processing procedure was performed for two reasons: (i) it allowed the subsequent ICA decomposition of the data to be simplified, because the great majority of the variance is concentrated on a reduced number of eigenvectors, meaning all the data can be explained in fewer components, and (ii) by doing so, we expect to filter out some of the background noise, which also facilitates the ICA decomposition (Shlens, 2014).

Spatial ICA was then applied to this dimensionally reduced data (FastICA v2.5, Espoo, Finland; Hyvarinen, 1999), allowing the linear superposition of signals that compose the *R*_2_* estimates to be separated into independent processes (or components). Each independent component has a (previously unknown) unique time-course and an associated spatial map, and can be studied individually. The decomposition process was modelled as:


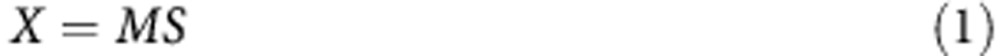


where *X* is the voxel-wise matrix of *R*_2_* time-courses, *M* is the ‘mixing' matrix, whose columns are filled with the component time-courses derived from the decomposition, and the rows of *S* contain the generated spatial maps. Separation between systemic and tumour-specific influences on tumour *R*_2_* fluctuations was undertaken by correlating each independent component time-course with the systemic O_2_sat time-course (Pearson's linear correlation). A significant correlation (*P*<0.01) suggested that the current independent component represented systemic blood oxygen dynamics. Conversely, if the correlation was less significant (*P*>0.01), the time-course of the independent component, its frequency spectrum and its spatial map were studied in order to assess if it was a component modelling tumour-specific dynamics. Two selection criteria were adopted: (i) if the frequency spectrum presented a peak at a defined frequency, this was enough evidence of cycling behaviour, or (ii) if the independent component spatial maps, which were normalised and thresholded by *z*-value (**|***z*-score**|**⩾2.2; van de Ven *et al*, 2004), presented clustering regions (minimum of 5 voxels), this meant that those regions shared a degree of similarity in their *R*_2_* dynamics. To improve the robustness of the second selection criteria, we only selected the components that exhibited spatial clustering regions associated with non noisy-like (distinctive) temporal features, in order to prevent noisy independent components from being considered (see Figure 1D). Any remaining components (i.e., ones with poor correlation and that did not exhibit a frequency peak or spatial clustering effects), were considered to be modelling any background noise not previously filtered by PCA and were not considered for further analysis.

Thus, this framework provides a method to potentially separate systemic from tumour-specific cycling hypoxia events in tumours. Because each component time-course has an associated component spatial map, we combined these two pieces of information to recover the *R*_2_* data, although now separated into systemic and tumour-specific influences, in a process which is the reverse of the ICA decomposition:


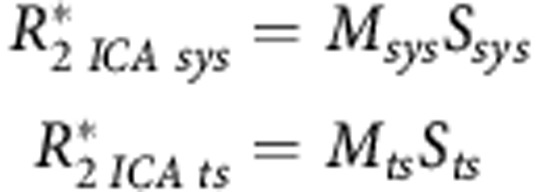


where 
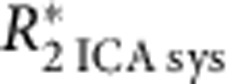
 and 
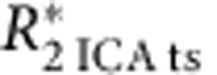
 are the voxel-wise matrices of systemic and tumour-specific *R*_2_* time-courses, respectively. Lastly, to threshold 
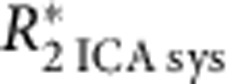
, a final correlation between the systemic O_2_sat and the time-courses of individual voxels of 
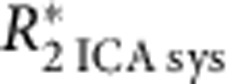
 was computed (Pearson's linear correlation). Only the most significant correlations (*P*<0.01) were selected to represent systemically oscillating tumour regions. A similar procedure was employed to detect the most tumour-specific fluctuating areas, where only non-significant correlations (*P*>0.05) between the systemic O_2_sat and the dynamics of 
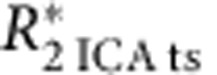
 were considered.

### Frequency analysis

Time-courses of thresholded 
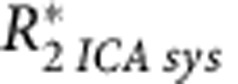
 and 
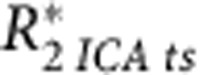
 were analysed in the frequency domain, for both tumour lines. For each tumour, individual voxel time-courses were Fourier transformed and subsequently combined into a single average-frequency spectrum. The spectral mean and standard deviation (s.d.) of the cohort of tumours was then computed. Dominant frequencies of oscillation were assessed based on spectral power (peak height).

### Histology and immunostaining

To investigate the pathophysiology underpinning fluctuations in BOLD-ICA measurements, we performed multifluorescence labelling of 10 *μ*m-thick frozen sections of tumour tissue. Following MRI, care was taken to preserve the orientation of the tumour upon excision and during histological preparation, so that tumour sections were cut in the same plane as the MRI acquisition. Histology could not be performed on one LS174T tumour due to sample degradation following excision. We obtained distributions of vascular perfusion, vascular endothelial cells, pericytes and hypoxic regions in all tumours. Perfusion staining was achieved via intravenous injection (12.5 mg kg^−1^) of the blue fluorescent marker Hoechst 33342 (Cambridge Bioscience, Cambridge, UK) 1 min before killing the animal. Endothelial cells were stained with a rat monoclonal antibody against CD31 (Abcam, Cambridge, UK), revealed with Alexa Fluor-488 (Life Technologies, Paisley, UK). Pericytes were detected by *α*-smooth muscle actin (*α*-SMA) staining with a mouse monoclonal anti-*α*-SMA (Sigma-Aldrich, Gillingham, UK), revealed with Alexa Fluor-546 (Life Technologies). Hypoxic regions were detected via intraperitoneal injection (60 mg kg^−1^) of the hypoxia marker pimonidazole (Hypoxyprobe, Burlington, MA, USA) 30 min before killing the animal, followed by *ex vivo* staining using a rabbit polyclonal anti-pimonidazole (Hypoxyprobe), revealed with Alexa Fluor-488 (Life Technologies). In addition, haematoxylin and eosin (H&E) staining was performed in immediately adjacent frozen sections to assess tumour morphology.

Fluorescence images were taken on an Axio Imager microscope (Carl Zeiss, Cambridge, UK), using an AxioCam digital colour camera. Images of H&E were collected on a bright-field Zeiss Axioskop2 microscope (Carl Zeiss). Both microscopes were fitted with a motorised stage and used AxioVision software (Carl Zeiss).

Quantification of immunofluorescence was performed in Matlab software (The MathWorks, Natick, MA, USA). Regions of interest (ROIs) were drawn around the total area of the tumour and images were thresholded to exclude background fluorescence signal. Tumour coverage of each of the four fluorescent markers was calculated as the ratio between fluorescent pixels and the total number of pixels within the ROI. In addition, the percentage of capillaries (CD31) covered with pericytes (*α*-SMA) was calculated to assess vascular maturation.

### Statistical analysis

Matlab software was used to perform statistical analysis. Assessment of linear correlation between O_2_sat and ICA time-courses was achieved by the Pearson's correlation coefficient and further transformed into a *t*-statistic to obtain *P*-values. For statistical comparisons between tumour size and other whole-tumour measurements, smaller sample numbers required the use of non-parametric statistics. Wilcoxon matched-pairs tests were used to compare between systemic and tumour-specific distributions. Mann-Whitney *U*-tests were used to assess differences between tumour types. Spearman's rho was used for correlation assessments. *P*<0.05 was considered to be significant, unless otherwise stated.

## Results

### Assessment of temporal and spatial variations in tumour R_2_*

Temporal fluctuations in *R*_2_* time-courses (derived from BOLD MRI measurements), which are thought to be caused by changes in deoxyhaemoglobin concentration, were observed in both LS174T and SW1222 tumour types. Example time-courses are shown in Figure 1B, alongside a map of the temporal s.d., which showed a heterogeneous distribution across the tumour. According to spectral analysis, the mean peak frequency in *R*_2_* time-courses was in the range inferior to 0.001 Hz (i.e.,<3.6 cycles h^−1^) for both tumour types. *R*_2_* values between SW1222 and LS174T tumours were 0.12±0.02 ms^−1^ and 0.08±0.02 ms^−1^ (mean±s.d.), respectively, which were not significantly different (*P*>0.05, Mann-Whitney *U*-test). However, as we are interested in temporal fluctuations, the relative variations in *R*_2_* assume greater importance than the actual *R*_2_* values. Moreover, it was shown that the absolute value of *R*_2_* (or *T*_2_*) does not reliably predict pO_2_ *in vivo*, although a good temporal correlation between these two quantities was observed (Baudelet and Gallez, 2002).

Decomposition of *R*_2_* time-courses using ICA produced, on average, 89 separate components; one component is shown in Figure 1D. Regional clustering was observed in the ICA spatial maps, which were thresholded by *z*-score (*z*-score<−2.2 or *z*-score>2.2), suggestive of spatial coherence in *R*_2_* fluctuation patterns (Figure 1D). This effect is often observed across several imaging slices, as demonstrated by the 3D representation, which suggests temporally coherent patterns over a tumour volume. The pathophysiological basis for such regions is discussed below.

### Relationship between systemic and tumour-specific influences on tumour *R*_2_* fluctuations

Alongside BOLD MRI data, pulse oximetry measurements were also acquired. An important aim of this study was to investigate, using ICA and pulse oximetry (ICA-O_2_sat), whether such tumour spontaneous fluctuations included contributions from systemic variations in blood oxygenation.

**Figure 2:**
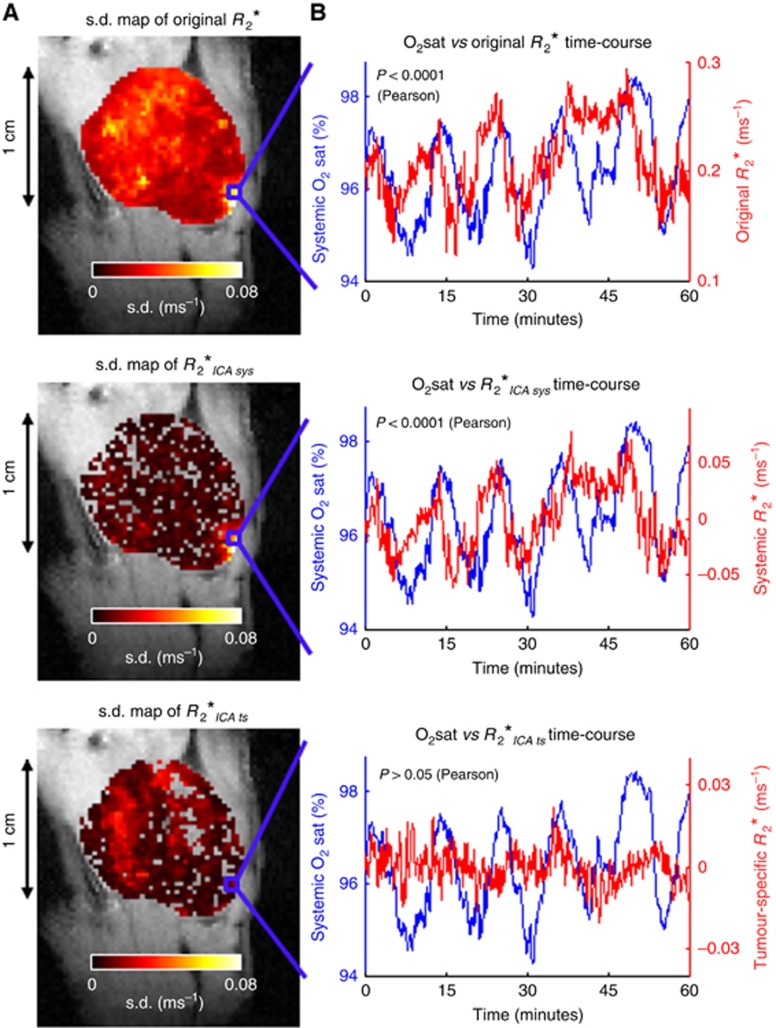
Systemic and tumour-specific influences on tumour *R*_2_* spontaneous fluctuations in a representative SW1222 tumour. (**A**) Voxel-wise s.d. maps of the *R*_2_* fluctuations before ICA decomposition (original *R*_2_*) and owing to systemic (
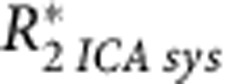
) or tumour-specific (
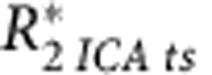
) influences, following ICA decomposition. High s.d. in different regions between both ICA maps distinguishes the tumour areas most affected by either source. Colocalised voxels between both ICA maps reflect the simultaneous influence of systemic and tumour-specific events in those tumour regions. (**B**) Correspondence between the O_2_sat curve (blue) and the *R*_2_* time-course from a single voxel (red) before ICA separation (top: original *R*_2_*) and after (middle: 
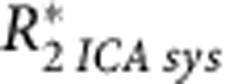
, bottom: 
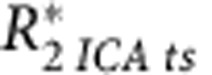
). O_2_sat and *R*_2_* estimates were acquired simultaneously.

Figure 2 shows an example SW1222 tumour, in which the ICA-O_2_sat model enabled the identification of *R*_2_* time-courses that were significantly correlated with systemic blood oxygen variations (*P*<0.01, Pearson's correlation; 
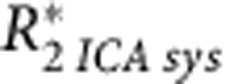
). In addition, we observed *R*_2_* temporal patterns that were not correlated with systemic blood oxygen variations (*P*>0.05, Pearson's correlation), which we considered to be specific to the tumour microenvironment (
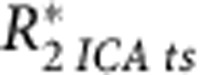
). These two types of oscillation could, however, be found in the same voxel and were not necessarily from discrete regions in the tumour. Both systemic and tumour-specific contributions were observed in all SW1222 (*n*=5) and LS174T (*n*=5) tumours.

**Figure 3:**
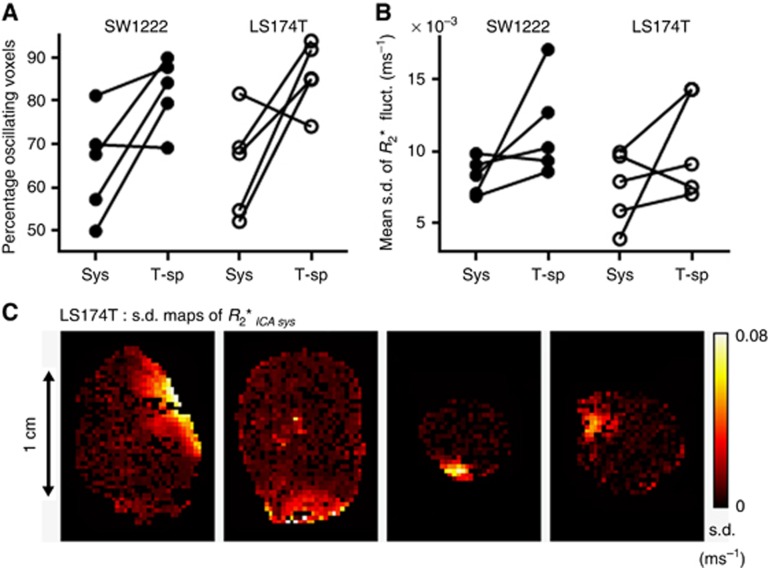
**Relative influence of systemic (Sys) and tumour-specific (T-sp) fluctuations in SW1222 and LS174T tumours and their spatial distribution.** (**A**) Percentage of systemic and tumour-specific oscillating voxels. (**B**) Mean s.d. of *R*_2_* fluctuations. In both tumour lines, fluctuations owing to tumour-specific influence showed higher prevalence and magnitude in 4 out of 5 tumours, relative to systemic fluctuations, although these differences were not statistically significant (Wilcoxon matched-pairs test). (**C**) Maps of 
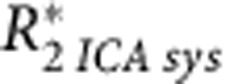
 of four LS174T tumours showing high amplitude systemic fluctuations in the tumour periphery. Conversely, SW1222 fluctuations (systemic and tumour-specific), and LS174T tumour-specific fluctuations, displayed no obvious spatial patterning.

Figure 3A shows the percentage of voxels exhibiting systemic or tumour-specific *R*_2_* fluctuations in each tumour line. The majority of SW1222 and LS174T tumours (4 out of 5 in each group) showed a greater occurrence of tumour-specific fluctuations than systemic fluctuations, although the difference was not statistically significant (both tumour lines: *P*=0.125, Wilcoxon matched-pairs test). A similar result was observed in the s.d. of *R*_2_* fluctuations (Figure 3B), in which tumour-specific fluctuations were of higher magnitude in all but one SW1222 or LS174T tumours (SW1222: *P*=0.125, LS174T: *P*=0.3125, Wilcoxon matched-pairs test).

All LS174T tumours exhibited large systemic fluctuations in the tumour periphery (Figure 3C), whereas tumour-specific fluctuations were observed in both the centre and the periphery. Conversely, systemic and tumour-specific fluctuations were distributed throughout SW1222 tumours.

**Figure 4:**
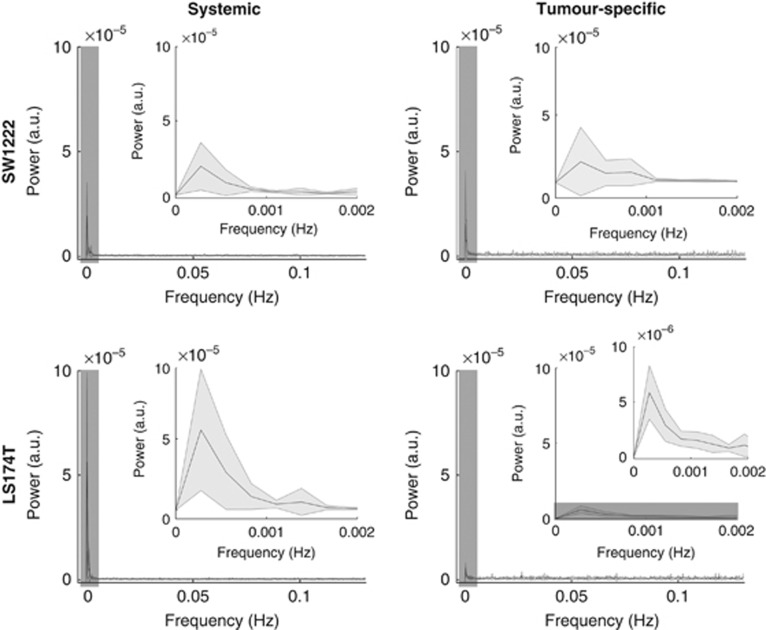
Frequency analysis of tumour *R*_2_* oscillations, organised by tumour line and systemic or tumour-specific influence. Mean (black line) and s.d. (shaded area) of power spectra are shown. Dominant frequencies of oscillation were found to be inferior to 1 × 10^−3^ Hz (<3.6 cycles  h^−1^) for all groups. Despite comparable frequencies, systemic and tumour-specific oscillatory sources are still separable. Inset graphs show magnifications of the shaded rectangles. One additional magnified spectrum is shown for the low-power LS174T tumour-specific oscillations.

Dominant frequencies for each type of fluctuation, assessed from the peak height of frequency spectra, were not significantly different in either tumour type, being both in a low-frequency range, inferior to 3.6 cycles h^−1^ (Figure 4). This result further demonstrates the discriminatory ability of the ICA-O_2_sat model, as similar frequencies were not a limiting factor in separating systemic and tumour-specific sources.

### Physiological basis of tumour fluctuations

**Figure 5:**
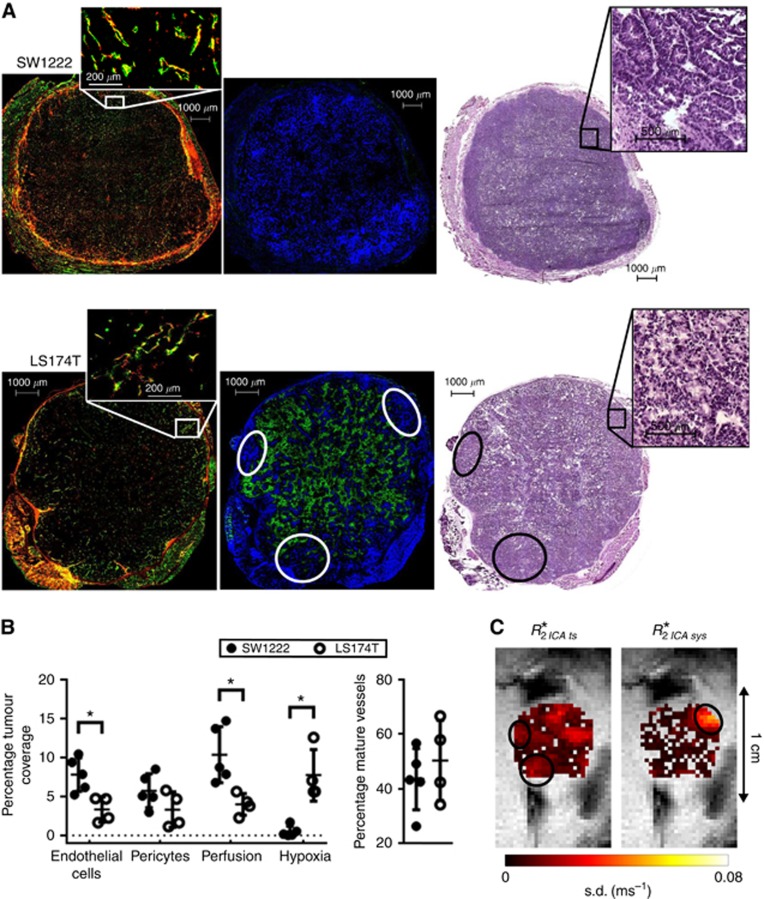
**Tumour histology for SW1222 (*n*=5) and LS174T (*n*=4) tumour xenografts and comparison with MRI data.** (**A**) Left: double staining for CD31 (endothelial cells, green) and *α*-SMA (mature vessels, red). Insets show blood vessels covered with pericytes (yellow). Middle: double staining for Hoechst 33342 (perfusion, blue) and pimonidazole (hypoxia, green). Right: morphology (H&E) showing moderate-to-well differentiated tissue into glandular structures in the SW1222 tumour and poorly differentiated tissue in the LS174T tumour. (**B**) Left: percentage tumour coverage of endothelial cells, pericytes, perfusion and hypoxia. Right: percentage of blood vessels covered with pericytes. Bars represent mean±s.d. SW1222 tumours have significantly higher vascular density and are better perfused and less hypoxic than LS174T tumours (Mann-Whitney *U-*test). No difference was found in the percentage of pericytes or in its coverage of blood vessels between both tumour types (Mann-Whitney *U-*test). (**C**) s.d. maps of tumour-specific and systemic *R*_2_* fluctuations (
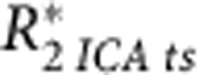
 and 
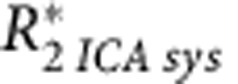
, respectively) corresponding to the LS174T tumour shown in panel **A**. Oval fiducial markers indicate association between elevated *R*_2_* s.d. and perfused vasculature. These areas also show viable (non-necrotic) tissue (H&E, see inset). SW1222 tumours show an identical relationship (see Figures 2A and 5A). *P<0.05.

Fluorescently labelled tumour sections revealed significant differences between tumour types, with SW1222 showing a more extensive vascular network (CD31), which was better perfused (Hoechst 33342) and less hypoxic (pimonidazole) than in LS174T tumours (Figure 5A and B). These observations are in accordance with previous studies (Pedley *et al*, 2002; El-Emir *et al*, 2005). Furthermore, visual inspection of H&E sections confirmed the different morphology of each tumour line, with SW1222 tumours showing better differentiation into glandular structures, which is in agreement with previous literature (Figure 5A; Folarin *et al*, 2010).

**Figure 6:**
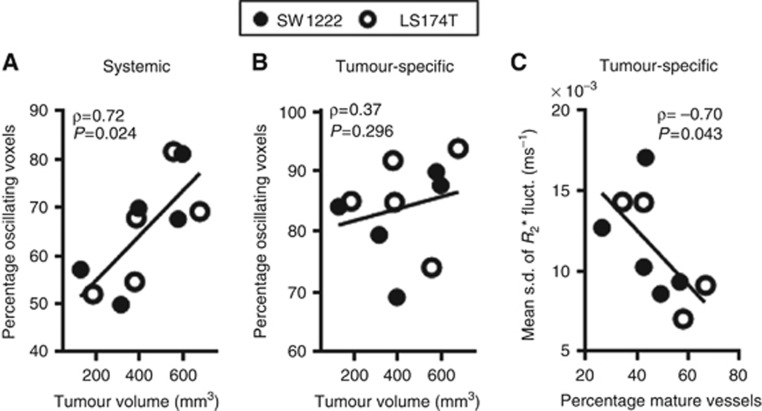
Relationship between tumour size, *R*_2_* fluctuations and mature vasculature. (**A**, **B**) Influence of tumour size on the occurrence of systemic or tumour-specific *R*_2_* fluctuations. The percentage of systemically oscillating voxels is directly related to the size of SW1222 and LS174T tumours (*P*=0.024, Spearman's ρ), but no relationship was found with tumour-specific oscillations (*P*=0.296, Spearman's ρ). (**C**) The percentage of mature vessels is inversely related to the mean s.d. of tumour-specific *R*_2_* fluctuations (*P=*0.043, Spearman's ρ).

The percentage of each tumour that exhibited *R*_2_* fluctuations due to systemic influences was significantly correlated with tumour volume (Figure 6A). There was no correlation between tumour volume and the percentage of each tumour exhibiting tumour-specific fluctuations (Figure 6B). However, comparison of histological measures with MRI data revealed a significant inverse correlation between the magnitude of tumour-specific fluctuations and the percentage of mature vessels (assessed by co-localisation of *α*-SMA and CD31 immunostaining, Figure 6C). No correlation was found between the percentage of mature vessels in each tumour and the size of the tumour, nor with the prevalence or magnitude of systemic fluctuations.

The percentage of pericytes, alongside the degree of attachment to tumour blood vessels, was not significantly different between the tumour types (Figure 5B). However, we observed that, in tumour sub-regions with high systemic or tumour-specific *R*_2_* variation, tissues were well perfused and viable (non-necrotic) in 4 SW1222 (*n*=5) and 3 LS174T (*n*=4) tumours (see Figure 5A and C for LS174T; see Figures 2A and 5A for SW1222).

## Discussion

In this study we have presented a novel technique, based on ICA, for characterising individual contributions to cycling hypoxia in tumours. Specifically, our approach was designed to separate systemic from tumour-specific sources of *R*_2_* variation (an MRI estimate of blood oxygenation) in individual tumours, whilst attempting to remove the influence of background noise inherent in the MRI acquisition process. This allowed the identification of the areas mostly affected by either mechanism in individual tumours. Low-frequency fluctuations in oxygen tension and blood flow were previously observed in both tumour and muscle by Braun *et al* (1999), using recessed-tip oxygen microelectrodes and laser-Doppler flow probes. Despite having observed greater low-frequency activity and greater magnitude changes in tumours, this provided an early suggestion for systemic influences. Baudelet *et al* (2004) also reported a similar observation when comparing *T*_2_*-weighted gradient-echo fluctuations between tumour and contralateral muscle. They additionally looked at whether muscle and tumour time series were correlated, and concluded systemic changes did not affect the tumour microenvironment because no significant results were obtained. The technique presented here is, however, more refined, as it isolates high-variance data and separates the multitude of processes contributing to *R*_2_* fluctuations before any time series correlation. Moreover, the ICA-O_2_sat model provides the unique advantage of being able to identify tumour regions that are simultaneously suffering from systemic and tumour-specific effects, which would be otherwise impossible, that is, if direct correlations were used without prior ICA decomposition.

We observed that systemically driven, high-amplitude fluctuations were preferentially located in the tumour periphery in LS174T tumours, whereas in SW1222 tumours there was no preferential location. This observation is potentially due to the less extensive vascular network evident in LS174T tumours, compared with SW1222 tumours. Fewer blood vessels can lead to impaired perfusion which, in combination with the higher microvascular density in the LS174T tumour periphery, potentially reduces the influence of systemic variations in the central area of the tumour. A similar observation was reported in an MRI study of A-07 melanoma xenografts, where blood flow fluctuations occurred preferentially in the tumour periphery, in which case the authors considered vasomotor activity in the supplying vasculature as a probable cause of fluctuations (Brurberg *et al*, 2007). Conversely, SW1222 tumours have a vascular tree closer to that of normal tissues, leading to greater perfusion throughout the tumour (and less hypoxia), and potentially allowing the transmission of systemic variations from the tumour edge towards its core more effectively. An important point to note in this regard is that, given the small percentage of hypoxic areas in SW1222 tumours, the observed *R*_2_* fluctuations in these tumours might not correspond to cycling hypoxia, but rather to oscillations in the blood oxygenation that remains within normal oxygenation levels. In this case, ‘cycling oxygenation' might be a more accurate term than ‘cycling hypoxia'.

Systemic and tumour-specific sources of *R*_2_* oscillations were further assessed by comparing the percentage of oscillating voxels with the tumour size. The occurrence of systemic, but not tumour-specific, *R*_2_* oscillations was found to be directly related to the size of tumours in both SW1222 and LS174T xenografts. Related to this, Chaplin *et al* (1986) had previously reported a relationship between the occurrence of cycling hypoxia and the size of SCCVII tumours. In their study, they injected the fluorescent perfusion marker Hoechst 33342 20 min before applying 10 Gy of radiation to tumours. Subsequently, a large differential survival between well-perfused cells and poorly perfused cells was observed in small tumours (≤200 mg). However, this difference decreased as the tumour size increased, which they inferred was due to a higher occurrence of cycling hypoxia in larger tumours, which acted as a protective mechanism against radiation. They suggested this relationship could also depend on tumour type and site of implantation. Given the results of the current study, we hypothesise that, as tumours develop and acquire an increased blood supply from the surrounding vasculature, the influence of systemic blood flow variations in the tumour becomes increasingly prevalent. Such effects can potentially occur by (i) an increase in the number of systemic feeding vessels accompanying tumour growth or (ii) an increase in the diameter of those vessels. Nonetheless, to the best of our knowledge, the evolution of the tumour feeding vasculature has not yet been directly investigated and a study of this nature would provide valuable information about the systemic influences on tumour cycling hypoxia.

Oscillations in *R*_2_* estimates additionally revealed a link to tumour biology. The amplitude of tumour-specific fluctuations was found to be inversely correlated to the degree of vessel maturation in SW1222 and LS174T tumours, but no similar relationship was found with systemic fluctuations. This illustrates a key benefit of the method developed in this study, in that isolation of tumour-specific fluctuations from those occurring owing to systemic influence allows a clear insight into tumour pathophysiology. Interpretation of this finding relies on the role of mural cells in the structural support of blood vessels: mature vessels have increased integrity and stability (Murakami, 2012), thus being less susceptible to mechanical stress-related microenvironmental factors responsible for cycling hypoxia, such as raised interstitial fluid pressure (Mollica *et al*, 2003). This interpretation is in agreement with a previous electron paramagnetic resonance imaging study of two carcinoma models, which suggested an inverse relationship between the maturity of the tumour microvascular network and the amplitude of tissue pO_2_ fluctuations (Yasui *et al*, 2010). A similar conclusion was reported in a related study of melanoma and cervical carcinoma xenografts, where the presence of connective tissue embedding the tumour microvessels was associated with stabilisation of blood flow and tissue pO_2_ fluctuations (Ellingsen *et al*, 2012). Moreover, *T*_2_*-weighted MRI estimates of pO_2_ and blood flow in syngeneic fibrosarcoma-II tumours previously showed that signal fluctuations occur predominantly in regions of immature vasculature (Baudelet *et al*, 2006). Vasomotion of mature vessels may still be an important fluctuating source in some tumour types (Martinive *et al*, 2006a).

The dominant oscillatory frequencies of R_2_* estimates reported in this study occurred at or below 1 × 10^−3^ Hz (<3.6 cycles h^−1^). These time scales are in the same range of the cycling hypoxia periods first reported by Chaplin and colleagues in a number of different preclinical tumour models (Chaplin *et al*, 1986; Minchinton *et al*, 1990). Using a fluorescence-activated cell sorting technique combined with radiation, they inferred cycles of hypoxia with a periodicity of 20–30 min. Almost two decades later, Cárdenas-Navia *et al* (2004) used recessed-tip oxygen microelectrodes to directly measure tumour pO_2_ fluctuations, which were observed to occur between 2 and 5 cycles h^−1^ in two different preclinical tumour lines. A number of other studies in different tumour lines reported fluctuating periods between 20 and 60 min (Dewhirst *et al*, 1996; Hill *et al*, 1996; Kimura *et al*, 1996). Less anticipated was the finding that systemic and tumour-specific *R*_2_* patterns oscillate in a similar frequency range. One possible issue has to do with the protocol used in this study. The sampling frequency of acquisition, 3.8 s per image (∼0.13 Hz), might not be fast enough to prevent signal interference from high-frequency oscillations. Indeed, Kalcher *et al* (2014) observed, using two different MRI signal acquisition rates, that the high-frequency proportions of signal fluctuations in the brain were folded into the low-frequency range when they used the low acquisition rate (∼0.25 Hz). This was evident upon comparison with spectral distributions of data acquired with a high rate (∼1.4 Hz). Conversely, scan times longer than those used in this study could help resolve any differences in the low-frequency range, either between systemic and tumour-specific sources or between tumour lines.

Besides the potential issues due to the relatively low-sampling rate of acquisition, the approach used in this study suffers from additional limitations. Estimating blood oxygenation variations with gradient-echo MRI can have confounding effects, as *R*_2_* changes can also reflect changes in blood volume, blood pH, haematocrit or in the fraction of oxygen extraction by the tissue (Howe *et al*, 2001). In addition, the ICA-O_2_sat model, as implemented here, did not consider any possible lag between the O_2_sat variations and tumour *R*_2_* fluctuations. However, the circulation time in mice is of the order of the temporal resolution of the GRE acquisition used in this study, so such effects can be assumed to be negligible. Finally, systemic influences in the tumour microenvironment can come from different sources, such as pulsatile flow and blood pressure, in addition to blood oxygenation, which might render the ICA-O_2_sat model an over-simplistic approach.

Nevertheless, the ICA-O_2_sat model is a non-invasive and novel approach to directly identify systemic influences on tumour cycling hypoxia patterns. We consider it has the potential to be easily translatable to the clinic, where tumour cycling hypoxia is still poorly characterised, with only a few published studies to date (Pigott *et al*, 1996; Nehmeh *et al*, 2008). If applied in the clinical setting, ICA-O_2_sat or its future versions would help predict the temporal and spatial delivery of systemically applied therapies, such as chemotherapy or radioimmunotherapy, in individual patients, and consequently help tailor a more effective delivery regime. In addition, measurements of fluctuations in oxygenation could potentially provide an insight into tumour pathophysiology in a clinical context, given the relationships found with vascular characteristics in this study.
