# Decomposition of spontaneous fluctuations in tumour oxygenation using BOLD MRI and independent component analysis

**DOI:** 10.1038/bjc.2016.140

**Published:** 2016-05-26

**Authors:** Miguel R Gonçalves, S Peter Johnson, Rajiv Ramasawmy, R Barbara Pedley, Mark F Lythgoe, Simon Walker-Samuel

**Correction to:** *British Journal of Cancer* (2015) **113**, 1168–1177; doi:10.1038/bjc.2015.270; published online 20 October 2015

**Updated online 26 May 2016:** This article was originally published under a CC BY-NC-SA 4.0 license, but has now been made available under a CC BY 4.0 license. The PDF and HTML versions of the paper have been modified accordingly.

